# 759 Comparing Burn Model System and ABA Registries: Differences in Sample Demographics and Burn Characteristics

**DOI:** 10.1093/jbcr/irad045.234

**Published:** 2023-05-15

**Authors:** Alyssa Bamer, Andrew Humbert, Kara McMullen, Gretchen Carrougher, Barclay Stewart, Nicole Gibran, Karen Kowalske, Jeffrey Schneider, Kyra Jeanine Solis, oscar Suman-Vejas, Haig Yenikomshian, Dagmar Amtmann

**Affiliations:** University of Washington, Golden, Colorado; University of Washington, Seattle, Washington; UT Southwestern Medical Center, Dallas, dallas, Texas; Spaulding Rehabilitation Hospital/Harvard Medical School/Rehabilitation Outcomes Center at Spaulding, Boston, Massachusetts; UT Southwestern Medical Center, Dallas, Texas; The University of Texas Medical Branch, Galveston, Texas; University of Southern California, Los Angeles, California; University of Washington, Seattle, Washington; University of Washington, Golden, Colorado; University of Washington, Seattle, Washington; UT Southwestern Medical Center, Dallas, dallas, Texas; Spaulding Rehabilitation Hospital/Harvard Medical School/Rehabilitation Outcomes Center at Spaulding, Boston, Massachusetts; UT Southwestern Medical Center, Dallas, Texas; The University of Texas Medical Branch, Galveston, Texas; University of Southern California, Los Angeles, California; University of Washington, Seattle, Washington; University of Washington, Golden, Colorado; University of Washington, Seattle, Washington; UT Southwestern Medical Center, Dallas, dallas, Texas; Spaulding Rehabilitation Hospital/Harvard Medical School/Rehabilitation Outcomes Center at Spaulding, Boston, Massachusetts; UT Southwestern Medical Center, Dallas, Texas; The University of Texas Medical Branch, Galveston, Texas; University of Southern California, Los Angeles, California; University of Washington, Seattle, Washington; University of Washington, Golden, Colorado; University of Washington, Seattle, Washington; UT Southwestern Medical Center, Dallas, dallas, Texas; Spaulding Rehabilitation Hospital/Harvard Medical School/Rehabilitation Outcomes Center at Spaulding, Boston, Massachusetts; UT Southwestern Medical Center, Dallas, Texas; The University of Texas Medical Branch, Galveston, Texas; University of Southern California, Los Angeles, California; University of Washington, Seattle, Washington; University of Washington, Golden, Colorado; University of Washington, Seattle, Washington; UT Southwestern Medical Center, Dallas, dallas, Texas; Spaulding Rehabilitation Hospital/Harvard Medical School/Rehabilitation Outcomes Center at Spaulding, Boston, Massachusetts; UT Southwestern Medical Center, Dallas, Texas; The University of Texas Medical Branch, Galveston, Texas; University of Southern California, Los Angeles, California; University of Washington, Seattle, Washington; University of Washington, Golden, Colorado; University of Washington, Seattle, Washington; UT Southwestern Medical Center, Dallas, dallas, Texas; Spaulding Rehabilitation Hospital/Harvard Medical School/Rehabilitation Outcomes Center at Spaulding, Boston, Massachusetts; UT Southwestern Medical Center, Dallas, Texas; The University of Texas Medical Branch, Galveston, Texas; University of Southern California, Los Angeles, California; University of Washington, Seattle, Washington; University of Washington, Golden, Colorado; University of Washington, Seattle, Washington; UT Southwestern Medical Center, Dallas, dallas, Texas; Spaulding Rehabilitation Hospital/Harvard Medical School/Rehabilitation Outcomes Center at Spaulding, Boston, Massachusetts; UT Southwestern Medical Center, Dallas, Texas; The University of Texas Medical Branch, Galveston, Texas; University of Southern California, Los Angeles, California; University of Washington, Seattle, Washington; University of Washington, Golden, Colorado; University of Washington, Seattle, Washington; UT Southwestern Medical Center, Dallas, dallas, Texas; Spaulding Rehabilitation Hospital/Harvard Medical School/Rehabilitation Outcomes Center at Spaulding, Boston, Massachusetts; UT Southwestern Medical Center, Dallas, Texas; The University of Texas Medical Branch, Galveston, Texas; University of Southern California, Los Angeles, California; University of Washington, Seattle, Washington; University of Washington, Golden, Colorado; University of Washington, Seattle, Washington; UT Southwestern Medical Center, Dallas, dallas, Texas; Spaulding Rehabilitation Hospital/Harvard Medical School/Rehabilitation Outcomes Center at Spaulding, Boston, Massachusetts; UT Southwestern Medical Center, Dallas, Texas; The University of Texas Medical Branch, Galveston, Texas; University of Southern California, Los Angeles, California; University of Washington, Seattle, Washington; University of Washington, Golden, Colorado; University of Washington, Seattle, Washington; UT Southwestern Medical Center, Dallas, dallas, Texas; Spaulding Rehabilitation Hospital/Harvard Medical School/Rehabilitation Outcomes Center at Spaulding, Boston, Massachusetts; UT Southwestern Medical Center, Dallas, Texas; The University of Texas Medical Branch, Galveston, Texas; University of Southern California, Los Angeles, California; University of Washington, Seattle, Washington; University of Washington, Golden, Colorado; University of Washington, Seattle, Washington; UT Southwestern Medical Center, Dallas, dallas, Texas; Spaulding Rehabilitation Hospital/Harvard Medical School/Rehabilitation Outcomes Center at Spaulding, Boston, Massachusetts; UT Southwestern Medical Center, Dallas, Texas; The University of Texas Medical Branch, Galveston, Texas; University of Southern California, Los Angeles, California; University of Washington, Seattle, Washington; University of Washington, Golden, Colorado; University of Washington, Seattle, Washington; UT Southwestern Medical Center, Dallas, dallas, Texas; Spaulding Rehabilitation Hospital/Harvard Medical School/Rehabilitation Outcomes Center at Spaulding, Boston, Massachusetts; UT Southwestern Medical Center, Dallas, Texas; The University of Texas Medical Branch, Galveston, Texas; University of Southern California, Los Angeles, California; University of Washington, Seattle, Washington

## Abstract

**Introduction:**

The Burn Model System (BMS) collects longitudinal data on burn survivors. This provides a unique resource for the burn research community, however, as patients are only enrolled in select sites across the United States, it is important to examine how similar the BMS participants’ demographics and burn characteristics are compared to the broader burn community. The purpose of this study was to compare burn survivors in the BMS to those in the American Burn Association’s (ABA) National Burn Registry (NBR).

**Methods:**

Demographic (age, sex, and race/ethnicity), injury (total burn surface area, etiology, presence of inhalation injury), and treatment (length of acute hospital stay, ventilator days) characteristics (as categorical variables) were compared between the BMS and ABA NBR for those with acute burn injuries from 2009 to 2014 who survived to hospital discharge. Differences and 95% confidence intervals were calculated for each category for adult and pediatric participants. Due to the large sample size and high power to detect non-meaningful differences, clinical significance was determined with the following criteria: a difference of >10% was major, 5-10% was minor, and < 5% was inconsequential.

**Results:**

A total of 1,080 BMS (765 adult; 315 pediatric) and 111,270 ABA NBR (77,528 adult; 33,742 pediatric) burn survivors were analyzed. Among adults, no notable differences were present for demographic characteristics. However, adults in the BMS had lower rates of injuries caused by scalding, higher rates of burn size over 15%, hospital stays more than 7 days, and any ventilator use. Similar trends were seen in children, however, BMS pediatric participants tended to be older, identified more frequently as Hispanic, and had higher rates of inhalation injury compared to the ABA NBR.

**Conclusions:**

Differences in demographic characteristics were inconsequential between the BMS and ABA NBR among adults. As expected, given the BMS inclusion criteria, BMS participants had more severe burns on average. Among children, major differences existed in many demographic, injury, and treatment characteristics. In part, these differences can be attributed to the difference in enrollment criteria between the databases and the over-representation of children from a single BMS site. Expanding the number of BMS centers and standardization of variables to allow for matching on inclusion criteria may improve the ability to compare the BMS with other samples.

**Applicability of Research to Practice:**

Due to differences in inclusion criteria, the BMS and ABA NBR differ in many patient characteristics.

